# Structure, Antioxidant, and Hypoglycemic Activities of Arabinoxylans Extracted by Multiple Methods from Triticale

**DOI:** 10.3390/antiox8120584

**Published:** 2019-11-25

**Authors:** Hong Chen, Zhuoyun Chen, Yuanfang Fu, Jiao Liu, Siying Lin, Qing Zhang, Yuntao Liu, Dingtao Wu, Derong Lin, Guoquan Han, Lina Wang, Wen Qin

**Affiliations:** 1 Department of Food Quality and Safety, College of Food Science, Sichuan Agricultural University, Yaan 625014, Sichuan, China; chenhong945@sicau.edu.cn (H.C.); sakiiiiis@163.com (Z.C.); yf18398265218@163.com (Y.F.); m15983089780@163.com (J.L.); linsiying132@163.com (S.L.); zhangqing@sicau.edu.cn (Q.Z.); lyt_taotao@163.com (Y.L.); DT_Wu@sicau.edu.cn (D.W.); lindr2018@sicau.edu.cn (D.L.); hans_980306@sicau.edu.cn (G.H.); 2 Department of Food Quality and Safety, Institute of Food and Drug Inspection, Chengdu 610000, Sichuan, China; calm945@aliyun.com

**Keywords:** arabinoxylans, extraction methods, structural features, antioxidant ability, hypoglycemic activity

## Abstract

Different methods of isolating arabinoxylans (AXs) from triticale were performed to investigate the extraction methods’ effects on the physiological functions of the AXs. Structural, antioxidant, and hypoglycemic activities were determined. The molecular weights (MWs) of enzyme- or water-extracted AXs were lower than those of alkali-extracted AXs. Opposite trends were shown by the arabinose–xylose ratio. Enzyme-extracted AXs exhibited higher glucose adsorption capacity and hydroxyl radical-scavenging efficiency than alkali-extracted AXs. The α-amylase inhibition ability, DPPH radical-scavenging capacity, and metal-chelating activity of alkali-extracted AXs were higher than those of enzyme-extracted AXs. Water-extracted AXs had the highest glucose dialysis retardation index. In conclusion, extraction methods can influence the physiological function of AXs through their structural features. AXs with higher MWs and esterified ferulic acid (FA) levels had higher antioxidant ability, whereas AXs with higher solubility and free FA level exhibited higher hypoglycemic activity.

## 1. Introduction

Arabinoxylans (AXs), which are non-starch polysaccharides derived mostly from cereals, demonstrate many physiological functions and health benefits. The primary structure of AXs is a backbone comprising β-1.4-d-xylopyranosyl with l-arabinofuranosyl substituted at position 2 and/or 3 of the furan cycle [1]. Ferulic acid (FA), which is the main hydroxycinnamic acid derivative in AXs, is linked to arabinofuranosyl residues through ester linkages with the 5-OH group [1].

AXs were reported to exhibit antioxidant ability. A lack of antioxidants in the human body results in oxidative stress, which is an imbalance between oxidants and antioxidants. Sufficient intake of dietary antioxidants is required to withstand oxidative stress, which leads to the prevention of type 2 diabetes, cardiovascular diseases, cancer, and Alzheimer’s disease [2]. Several research studies suggest that AX structure is related to their antioxidant activity [3,4,5]. Low-branched hydroxycinnamic acid-esterified AXs derived from kodo millet reportedly exhibit stronger antioxidant activity than AXs derived from four other kinds of India millets with relatively high degree of substitution (DS) [6]. Bijalwan et al. suggested that the abovementioned findings were due to the increase in antioxidant capacity induced by low-branched AX moieties [6]. Malunga et al. also proposed that FA content was the major factor that determines antioxidant capacity of arabinoxylooligosaccharides (AXOS) [4].

Aside from antioxidant ability, hypoglycemic activity is also a physiological function of AXs. One of the mechanisms is to decrease the postprandial hyperglycemia, which is induced by delaying glucose absorption owing to AX viscosity. The molecular structure effects of AX on viscosity have been reported in several studies that involve molecular weight (MW), DS, and FA content of AXs [1,7,8]. Moreover, AXs inhibit α-amylase and α-glucosidase in the digestive tract through their FA content [9,10]. Inhibition of α-amylase and α-glucosidase leads to the delay of increase in postprandial blood sugar levels. Additional studies have reported that antioxidant activity contributed to the ability of AXs to regulate glucose metabolism. The α-glucosidase and glucose transporter inhibition of AXs was possibly related to the antioxidant ability of FA in both free and esterified forms [10]. Zhang et al. further believed that the antioxidant capacity of AXs can reduce postprandial oxidative stress that was associated with lower risk of diabetes [11].

Hexapod triticale, which is a crossbreed between wheat and rye, serves as an important black food resource and exhibits significant antioxidant, antihypertensive, and cancer prevention effects [12]. High amounts of non-starch polysaccharides, anthocyanin, and phenolic acid of triticale contribute to such effects, which make triticale significant in AX extraction [13]. Water-, alkali-, and enzyme extraction, physical treatment, and their combination are applied to extract AXs from the sources [14]. The use of alkaline solutions is efficient for AX extraction from cell wall materials. However, the violent condition may break down ester linkages between arabinofuranosyl residues and hydroxycinnamic acid, thereby decreasing hydroxycinnamic acid content such as FA [15]. Enzyme extraction is regarded as a simple method to obtain AXs. This process can prevent some functional groups of AXs such as FA from separating and lowering the MW of AXs [15]. AX structure can be influenced by the application of different extraction methods. Likewise, structural changes can have an impact on physiological AX functions, such as antioxidant activity and hypoglycemic activity. Therefore, in the present study, we assumed that the extraction method plays a significant role in the change in AX physiological function via exerting effects on their structural features.

Currently, the impact of different extraction methods on AX physiological functions is still unclear. In this article, various extraction methods were applied to obtain AXs with different structural features. We combined enzyme extraction and ultrasonic-assisted extraction to obtained AXs from triticale. The combination of xylanase and cellulose was chosen for enzyme extraction. Alkali- or water-extraction methods were also applied to isolate AXs. Moreover, through alkali extraction, we acquired AXs from the residue of water and enzyme extraction. Structural and physiological functions of all AXs extracted through different methods were determined in this study. Based on these results, we preliminarily obtained the relationship between AXs’ structural features and physiological functions. This study also analyzed the influence of extraction methods on AXs’ physiological functions.

## 2. Materials and Methods

### 2.1. Materials

Triticale was obtained from the Hongbing ecological farm in Shandong, China. The triticale was milled and passed through a 0.425 mm sieve. Sunzymes that contained endo-1,4-β-xylanase and cellulose were obtained from Ruiyong Biotechnology Co., Ltd. in Shanghai, China. Neutral protease, glucoamylase, and α-amylase were also obtained from Ruiyong Biotechnology Co., Ltd. All solvents, chemicals, and reagents were of analytical grade and purchased from the market. 

### 2.2. AX Extraction

The activities of endogenous cell wall-degrading enzymes (e.g., endogenous arabinoxylanase) of triticale were destroyed using a drum wind dryer (HASUC, Shanghai, China) in 110 °C for 90 min. Afterwards, triticale was destarched and deproteinized and subsequently incubated at 60 °C for further extraction. 

Enzyme extraction of AXs from destarched and deproteinized triticale was performed in a 250 mL stoppered Erlenmeyer flask with 100 mL of 50 mmol/L sodium acetate buffer that contained the required amount of complex enzyme (i.e., combination of xylanase and cellulose). A total of 5 g of destarched and deproteinized triticale was added to the freshly prepared enzyme solution. The mixture was directly sonicated in an ultrasonic bath (Kunshan Ultrasonic instruments, Kunshan, China) at 150 W for 60 min. After heat inactivation of the enzyme at 90 °C for 15 min, the mixture was centrifuged to obtain the AX solution. The acquired supernatant was first concentrated to about one-fourth of the initial volume, then precipitated by ethanol, and subsequently centrifuged. The deposit was dissolved in distilled water and centrifuged. The solution was then purified. The solution was treated with α-amylase and protease again and was concentrated and precipitated by ethanol. The sediments were dissolved in water and centrifuged. The supernatant was precipitated by ethanol as mentioned above and freeze dried in a freeze dryer system (Heto PowerDry PL3000, Thermo Fisher Scientific, Shanghai, China). The obtained fraction was complex enzyme-extracted AXs (CEAXs).

The residue of enzyme extraction was further treated by alkali extraction. Extraction was done based on the procedure described by Zhou et al. in 2010 [15]. The residue was initially dried for 12 h at 60 °C. Afterwards, 0.16 mol/L NaOH (including 0.5% H_2_O_2_, *v*/*v*) was blended with the dried residue for 90 min at 80 °C. The mixture was subsequently cooled to room temperature and centrifuged. The supernatant was acidified to about pH 4.5 and centrifuged. The acquired supernatant was concentrated to about one-fourth of the initial volume, then precipitated using ethanol, and subsequently centrifuged. The deposit was dissolved in distilled water and centrifuged. The supernatant was purified and freeze dried. The obtained fraction was alkali-extracted AX from the residue of complex enzyme extraction (CEAX-1).

Destarched and deproteinized triticale was also treated by alkali extraction. The extraction procedure was similar to that used in residue extraction. The obtained fraction was alkali extractable AXs (AEAXs).

Water extraction was applied to obtain AX from triticale. About 5 g of destarched and deproteinized triticale were mixed with 100 mL distilled water and water bathed for 2 h at 60 °C followed by being cooled to room temperature and centrifuged. The supernatant was dialyzed, concentrated, and precipitated by ethanol. The sediments were dissolved in water and centrifuged. The supernatant was purified and freeze dried. The obtained fraction was water extractable AXs (WEAXs). The residue of water extraction was further treated by alkaliextraction. The obtained fraction was alkali-extracted AX from residue of water extraction (WEAX-1).

### 2.3. Monosaccharide Composition

A 10 mg sample of the AXs was mixed with 5 mL of 2 mol/L trifluoroacetic acid in a hydrolysis tube, and the mixture was incubated at 95 °C for 10 h in a heating block (Thermo Fisher Scientific, Shanghai, China). Trifluoroacetic acid was removed at 70 °C under a steady stream of nitrogen. The AX sample and monosaccharide standard derivatization were prepared as follows: all the AX samples (10 mg) or monosaccharide standards (2 mg) in each tube were mixed with 30 mg of hydroxylamine hydrochloride and 1.5 mL of pyridine, respectively. The tubes were incubated at 90 °C in a water bath shaker (Guowang, Changzhou, China) for 30 min and then cooled to room temperature. About 0.5 mL of acetic anhydride was added and mixed completely with the solution, and the tubes were then incubated in a water bath shaker in 90 °C for 30 min. About 0.1 mL of clear supernatant was added to autosampler vials after cooling with inserts for injection into a gas chromatograph. A Hewlett Packard chromatograph (5890, Palo Alto, CA, USA) coupled to a mass spectrometer was used to analyze derivatized arabinoxylan samples and monosaccharides. The 1 μL sample was introduced in the splitless injection mode onto an SP 2330 column (30 m × 0.25 mm, 0.25 μm film thickness, Supelco, Beijing, China) using helium as carrier gas. The derivatives were separated using the following temperature gradients, that is, 185 °C at 5 °C/min, 200 °C at 4 °C/min, and 280 °C at 20 °C/min, and such processes were conducted for 2 min. Samples were ionized by electron impact at 70 eV [16].

### 2.4. MW Determination

MW distribution of AXs extracted by different methods was determined by SE-HPLC at 38 °C. The 0.1 mol/L LiNO_3_ filtered through 0.2 μm (Whatman, (Qiaochen, Shanghai, China) was used to isocratic elution at 0.6 mL/min. MWs were determined after universal calibration with pullulans as standards (P50 to P800). About 20 μL of AX solution (0.5% *w*/*v*) filtered through 0.2 μm (Whatman) were injected and detected by a Waters 2414 refractive index detector (Agilent, Beijing, China) [17].

### 2.5. Infrared Spectroscopic Analysis

A Nicolet FT-IR spectrophotometer (Nicolet Instrument Corp., Madison, WI, USA) was applied to record FT-IR spectra of AXs extracted by different methods. The samples were pressed into KBr pellets (2 mg sample/200 mg KBr). A blank KBr disk was used as background. Spectra were recorded between 400 and 4000 cm^−1^ [17].

### 2.6. Determination of Free or Esterified FA Content

A spectrophotometric method based on the study of Malunga and Beta in 2015 [3] was applied to measure the level of free or esterified FA. Molar absorption coefficients (M^−1^cm^−1^) of 19,662 (7630) and 23,064 (31,430) for free FA and esterified FA were used to estimate the absorbance at 345 (375) nm, respectively. About 1 mL distilled water was added to dissolve 1 mg of AX sample. Then, a 100 µL portion of solution was mixed with 900 µL of glycine-sodium hydroxide buffer (pH 10, 0.04 mol/L). After 5 min, the absorbance value of mixture was determined (Ultrospec 1100 Pro, UV/Visible spectrophotometer, (Optima, SP-3000, Tokyo, Japan) at 345 and 375 nm.

### 2.7. Measurement of Hypoglycemic Activity of AXs

#### 2.7.1. α-Amylase Inhibition Assay

A mixture of 500 μL AXs or acarbose and 500 μL of 0.02 mol/L sodium phosphate buffer (pH 6.9 with 0.006 mol/L sodium chloride) containing α-amylase solution (13 U/mL) was incubated at 25 °C for 10 min. After preincubation, 500 μL 1% soluble starch solution in 0.02 mol/L sodium phosphate buffer (pH 6.9 with 0.006 mol/L NaCl) were added to each tube at timed intervals. The reaction mixtures were then incubated at 25 °C for 10 min, followed by addition of 1 mL dinitrosalicylic acid color reagent. The test tubes were then placed in a boiling water bath (Zhongxing, Beijing, China) for 5 min to stop the reaction and were cooled to room temperature. The reaction mixture was then diluted with 10 mL distilled water, and absorbance was read at 540 nm [18].

(1) $$\mathrm{inhibition}\left(\%\right)=\left(\frac{\Delta Abs_{control}-\Delta Abs_{sample}}{\Delta Abs_{control}}\right)\times 100\%$$

where $\Delta Abs_{control}$ and $\Delta Abs_{sample}$ are the absorbance of sample and control respectively.

The inhibitory activity was expressed as the half maximal inhibitory concentration (IC_50_), which is a measure of the effectiveness of a compound in inhibiting biological or biochemical function.

#### 2.7.2. Determination of Glucose Absorption Capacity

About 1 g of sample was added into 100 mL glucose solution (100 mmol/L, pH 7.0), and the mixture was shaken at 37 °C for 6 h. After centrifugation at 3000 r/min for 20 min, the glucose content in the supernatant was measured to estimate the amount of glucose adsorbed by the sample. The system without sample served as control test [19].

(2) $$\mathrm{adsorption}\;\mathrm{quantity}\left(\mathrm{mg}/g\right)=\frac{A_{control}-A_{sample}}{M_{sample}}\times 100\%$$

where *A_sample_* and *A_control_* are the absorbance of sample and control respectively, *M_sample_* is the weight of the sample.

#### 2.7.3. Glucose Dialysis Retardation Index (GDRI)

About 0.50 g of sample was added into 15 mL of glucose solution (100 mmol/L), and the mixture was shaken for 1 h. A 20 cm long dialysis bag (Nanjing SenBeiJia Biological Technology, Nanjing, China) (7000 MWCO) was filled with the mixture. The control was made using a mixture of deionized water and glucose solution. The bag was tied and suspended in 200 mL deionized water and placed in a stirred bath at 37 °C for 90 min. At 30, 60, and 90 min, 2 mL were taken from the dialysate and were analyzed for glucose content [19].

The GDRI is calculated as follows:

(3) $$\mathrm{GDRI}\left(\%\right)=\left(1-\frac{\mathrm{total}\;\mathrm{glucose}\;\mathrm{diffused}\;\mathrm{from}\;\mathrm{sample}}{\mathrm{total}\;\mathrm{glucose}\;\mathrm{diffused}\;\mathrm{from}\;\mathrm{control}}\right)\times 100$$

### 2.8. Estimation of Antioxidant Activity of AXs

#### 2.8.1. Hydroxyl Radical-Scavenging Assay

The abilities of AX samples in scavenging hydroxyl radicals were studied. Amounts of about 0.1mg/mL to 2.0 mg/mL of samples were dissolved in ultrapure water. A 1 mL of sample solution was mixed with 2 mL of reaction buffer (0.02 mol/L phosphate buffer (PBS), pH 7.4), 1.5 mL of 1,10-phenanthroline (1 mmol/L), 1 mL of FeSO_4_ (1.5 mmol/L), 1 mL of H_2_O_2_ (0.1%), and 3.5 mL of ultrapure water to form the mixture, which was incubated at 37 °C for 60 min. The existence of hydroxyl radical was determined by monitoring absorbance at 510 nm using a spectrophotometer, and the Vc was used as the positive control [20].

The scavenging activity of hydroxyl radicals was calculated via the following formula:

(4) $$\mathrm{scavenging}\;\mathrm{activity}\left(\%\right)=\frac{A_{2}-A_{1}}{A_{0}-A_{1}}\times 100$$

where *A*_1_ is the absorbance of ultrapure water instead of the sample, *A*_2_ is the absorbance of the polysaccharide sample, and *A*_0_ is the absorbance of the ultrapure water instead of the polysaccharide sample and H_2_O_2_.

#### 2.8.2. 2,2-Diphenyl-1-picrylhydrazyl (DPPH)-Scavenging Assay

Amounts of about 1.5 mL of AXs (0.1, 0.2, 0.4, 0.6, 0.8, 1.0, and 1.2 mg/mL) were mixed with 0.5 mL absolute ethyl alcohol followed by 0.5 mL DPPH (0.2 mmol/L DPPH-aqueous methanol). The mixtures were vortexed and remained in the dark for 35 min, and then the absorbance was measured at 515 nm. Ethanol instead of DPPH was used for the control, whereas distilled water instead of the sample was used for the blank [21]. The DPPH radical-scavenging activity of the sample was calculated by the following equation:

(5) $$\mathrm{DPPH}\;\mathrm{radical}\;\mathrm{scavenging}\;\mathrm{activity}\left(\%\right)=\left(1-\frac{A_{sample}-A_{control}}{A_{blank}}\right)\times 100\%$$

where *A_sample_*, *A_control_* and *A_blank_* are the absorbance of sample, control, and blank, respectively.

#### 2.8.3. Reductive Ability

Amounts of about 0.2 mL of samples (0.1, 0.2, 0.4, 0.6, 0.8, 1.0, and 1.2 mg/mL) in water were mixed with phosphate buffer (1.0 mL, 0.2 mol/L, pH 6.6) and potassium ferricyanide (K_3_Fe(CN)_6_) (1.0 mL, 1%). The mixture was incubated in a water bath at 50 °C for 20 min. A portion (1.0 mL) of trichloroacetic acid (10%) was added to the mixture, which was then left standing at room temperature for 10 min. The upper layer of solution (2.5 mL) was mixed with distilled water (1.0 mL) and FeCl_3_ (0.2 mL, 0.1%), and the absorbance was measured at 700 nm after 10 min against the blank. Vc with the same concentration served as the positive control [22].

#### 2.8.4. Metal-Chelating Activity

The samples (0.1 mg/mL to 2.0 mg/mL) were mixed with a 2 mmol/L FeCl_2_ solution (0.02 mL). The reaction started after the addition of 5 mmol/L ferrozine (0.02 mL). Subsequently, the mixture was shaken vigorously and left standing at room temperature for 10 min. Absorbance of the solution was then determined spectrophotometrically at 562 nm. EDTA disodium salt dihydrate (EDTA-Na_2_-2H_2_O) was used for the positive control [23].

### 2.9. Statistical Analysis

The mean ± standard deviation values of three replicates were applied to express the results. All data were statistically analyzed using SPSS (version 22.0 for Windows, SPSS Inc., (Chicago, IL, USA) with one-way analysis of variance (ANOVA) procedure, and then Tukey’s test was conducted. A *p*-value of less than 0.05 was considered to be significant.

## 3. Results and Discussion

### 3.1. Isolation and Characterization of AXs

The yield of AXs extracted through different methods is shown in Table 1. The yield of alkali-extracted AXs was higher than that of enzyme-extracted AXs and WEAX, which was consistent with previous reports [15]. Therefore, efficiency of alkaline solution was higher than that of complex enzyme in AX extraction. Moreover, the total yield of CEAX and CEAX-1 was higher than that of AEAX, which indicated that alkali extraction can be more efficient after enzyme extraction, thereby elevating total AX yield.

Monosaccharide compositions of triticale-extracted AXs were measured by HPLC, and the results are shown in Table 2. AXs extracted using different methods all contained nearly 90% of arabinose and xylose. Likewise, alkali-extracted AXs were highly substituted with an arabinose–xylose ratio of 1.14 to 1.52, which was higher than enzyme-extracted AXs with an arabinose–xylose ratio of 0.25. A previous study determined the monosaccharide compositions of AXs derived from wheat bran by enzyme, water, and alkali extraction [24]. The results also demonstrated that the arabinose–xylose ratio of alkali-extracted AXs was higher than that of enzyme-extracted AXs. Other minor neutral sugars, such as galactose and mannose, were detected in AXs, which was consistent with previous research studies [25,26].

MW distribution was determined (see Table 3). The MWs of enzyme-extracted AXs were lower than those of alkali-extracted AXs, consistent with a previous report [15]. The lower MW of enzyme-extracted AXs may be due to the ability of endo-1,4-β-xylanases to randomly cleave the xylan chain, which releases fractionated AXs and/or AXOS of various sizes [27].

**Figure 1:**
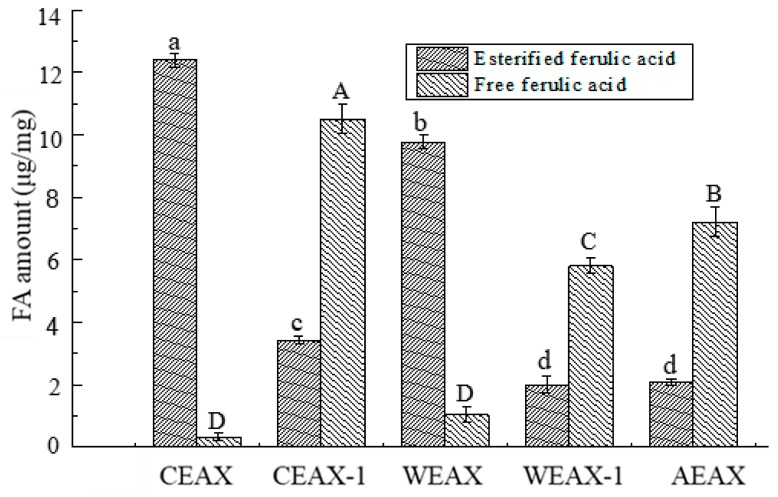
Amounts of esterified ferulic acid and free ferulic acid in arabinoxylans. “a–d” indicates a significant in longitudinal, and “A–D” represents a significant in lateral.

The content of free and esterified FA in AXs was determined, and the results are shown in Figure 1. Enzyme-extracted and water-extracted AXs had significantly higher esterified FA content than free FA content (*p* < 0.05). Malunga and Beta also observed that the amount of esterified FA was higher than free FA in enzyme-extracted AXs, which was in agreement with our result [3]. Likewise, alkali-extracted AXs had significantly higher free FA content than esterified FA content (*p* < 0.05). Alkali solutions are extremely violent and may break down ester linkage between FA and arabinofuranosyl residues; thus, a large amount of free FA was contained in alkali-extracted AXs. Furthermore, among all AXs extracted by different methods, CEAX-1 contained the highest amount of total FA and free FA, whereas CEAX contained the highest amount of esterified FA. 

Interestingly, our results demonstrated that the arabinose–xylose ratio and MW of CEAX-1 was similar to AEAX, and the amount of total FA of CEAX-1 was even higher than of AEAX. These results were obtained probably because the enzyme treatment increased the efficiency of subsequent alkali extraction. Alkali treatment on residues after enzyme extraction allowed for the full use of raw materials.

**Figure 2:**
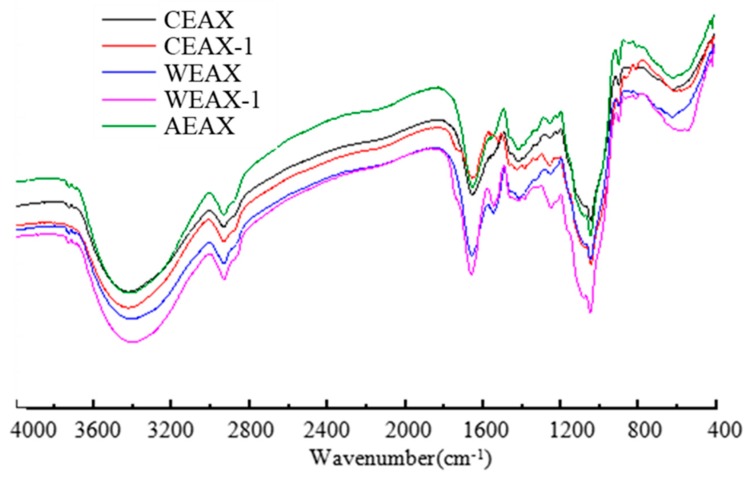
Fourier-transform infrared spectra of arabinoxylans.

The infrared absorption spectrum of AXs is shown in Figure 2. A wide polysaccharide absorption peak in the range of 1200 cm^−1^ to 800 cm^−1^, a stretch vibration peak of the glycosidic bond at 857 cm^−1^, a stretch vibration peak of methyl C–H at 2928 cm^−1^, and a bending vibration peak of C–H at 1409 cm^−1^ were observed. The wide peak at 3401 cm^−1^ was produced by the stretching vibration of the hydroxyl group; the strong peak at 1653 cm^−1^ resulted from the asymmetric stretching vibration of C–O in the carbonyl group; and the peak at 1039 cm^−1^ is attributed to the deformation vibration of alcohol hydroxyl O–H. The infrared absorption spectrum confirmed that the structural characteristics of the products extracted from triticale were consistent with AXs. Moreover, all absorption peaks had discrepancies in different AXs, which suggests that the structure of AXs extracted through different methods is inconsistent.

Based on the AX structural characteristics, antioxidant and hypoglycemic activities of AXs were estimated in further experiments, aiming to determine the relationship between AX structure and physiological functions.

### 3.2. Antioxidant Activity of AXs

**Figure 3:**
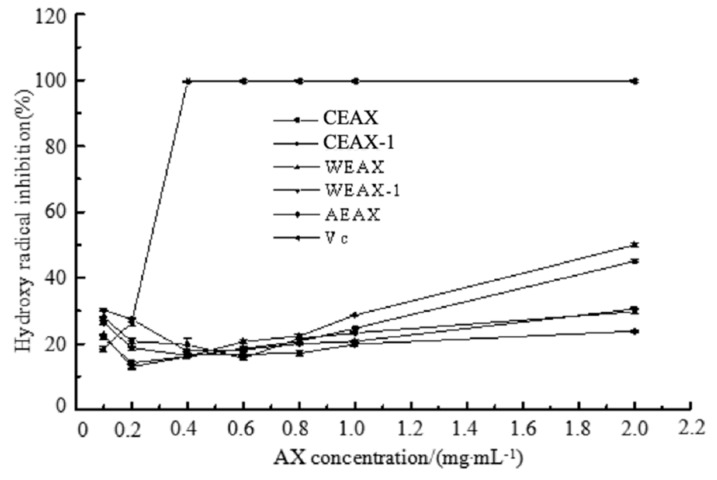
Hydroxyl radical-scavenging effects of arabinoxylans.

Our results showed that along with the increased AX concentration, the hydroxyl radical, DPPH radical-scavenging ability, and metal-chelating activity of AXs increased; and these results confirmed the antioxidant activity of AXs. AXs are capable of donating electrons or hydrogen atoms to free radicals, which leads to the transformation from free radicals to stable products [28,29]. Therefore, free radicals are eliminated, and oxidation reactions are interrupted. It has been reported that AX structure contributed to their antioxidant ability. Hydroxyl groups of AXs are the main structural features that affect their antioxidant capacity [30]. The presence of electrophilic groups in AXs helps to release hydrogen from O–H bonds. Our findings showed that the structural characteristic of the AX moiety should not be ignored when evaluating the AX antioxidant. Figure 3 shows the results of the hydroxyl radical-scavenging assay, which is based on the reduction of hydroxyl radicals in the existence of an electron-donating substance [31]. Based on the comparison between CEAX-1 and CEAX, CEAX exhibited higher hydroxyl radical-scavenging activity. Both CEAX and CEAX-1 contained similar amounts of FA, but CEAX had lower DS. Thus, lower DS contributes to higher antioxidant ability of CEAX. The unsubstituted xylose at O-2 and/or O-3 may have contributed to antioxidant capacity of AXs via donation of electrons or hydrogen atoms [30]. Low DS increases the specific functional groups of xylan to elevate hydroxyl radical-scavenging activity through donation of electrons or hydrogen atoms [32]; thus, the antioxidant activity of AX with low DS increased. 

**Figure 4:**
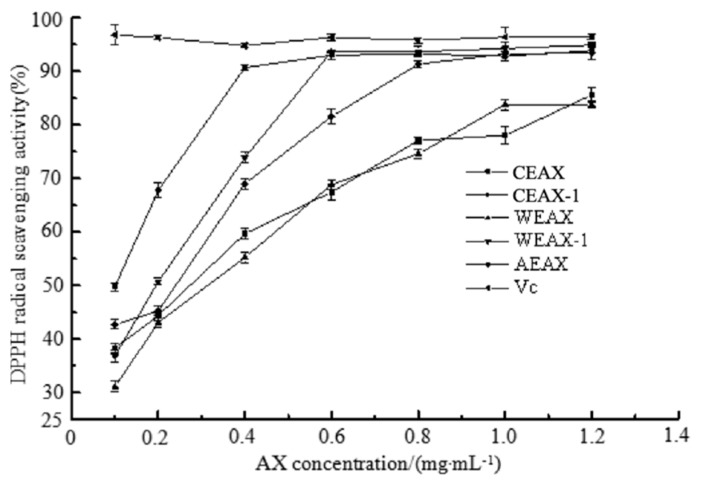
DPPH radical-scavenging effects of arabinoxylans.

DPPH radical-scavenging activity was measured in Figure 4, whose reaction mechanism involves the transfer of protons by the reducing agent to the DPPH radical [3,33]. In contrast, the DPPH radical-scavenging ability of AXs elevated in line with the increase in DS. This finding was inconsistent with the results of hydroxyl radical-scavenging assay. AXs with higher DS were found to be easier to disperse into the reaction mixture because the steric hindrance caused by the presence of mono- or disubstituted xylose prevented intermolecular cross-linking, which leads to better participation of AXs in the redox reaction systems [4]. Compared to the results of hydroxyl radicals with the DPPH radical-scavenging assay, we found that the structure of AXs would not only influence the amount of special functional groups, but also affect the interaction between AX molecules as well as between AXs and the solvent. In addition, both of these substances would exert an effect on antioxidant capacity. 

**Figure 5:**
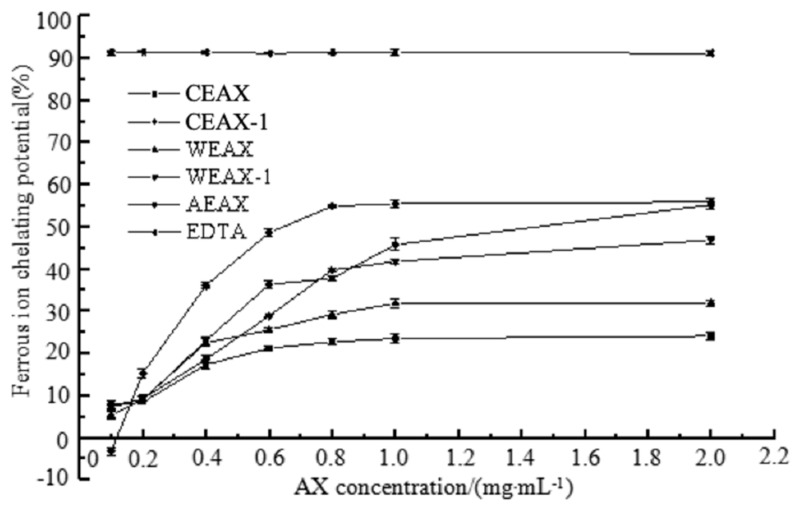
Metal-chelating activity of arabinoxylans.

The metal-chelating activity of AXs was determined in the present study (see Figure 5). It has been reported that the specific functional groups such as –COOH and –OH of AXs demonstrated the ability to bind metal ions when those functional groups were deprotonated and carried negative charges [34]. The metal-chelating activity of AX samples was elevated when the MW of the AXs was increased, despite the different FA content in the AXs extracted by different methods. Therefore, our results showed that AXs with higher MW lead to higher metal-chelating activity. High MW may elevate (1–4)-β-d-xylopyranose linkages to some extent, which leads to a higher amount of specific functional groups (such as –COOH and –OH) of xylan with metal-binding activity [32].

Moreover, we determined whether the FA content of AXs influenced their antioxidant ability. AEAX and CEAX-1 have similar MW and DS, but CEAX-1 performed higher hydroxyl radical- and DPPH radical-scavenging activity compared with AEAX. These results occurred because CEAX-1 contained a higher amount of FA, which suggested that FA content was the major determining factor for the antioxidant capacity of AXs [6]. Furthermore, among AEAX, CEAX-1, and CEAX, CEAX had better hydroxyl radical-scavenging ability. Hydroxyl radical-scavenging assay was performed in aqueous medium, and FA solubility in aqueous medium increased when FA was esterified to arabinose. Thus, esterified FA had a higher antioxidant capacity when compared with a similar amount of free FA in aqueous medium [2]. CEAX contained a higher amount of esterified FA than AEAX and CEAX-1, which may also be one of the reasons for higher antioxidant ability.

On the contrary, the result of DPPH radical-scavenging ability of AXs demonstrated that the antioxidant capacity of AEAX and CEAX-1 was higher than that of CEAX, although the latter contained higher esterified FA. It has been reported that in the presence of DPPH reagent, AXs with high esterified FA content may be oxidatively coupled in a phenomenon called oxidative gelation [4]. This phenomenon has been brought up previously [35]. Oxidative gelation decreases the potential of AXs to scavenge free radicals [4]. Since CEAX contained the highest esterified FA content among all samples, the FA residue of CEAX probably underwent oxidative gelation via cross-linking, which then reduced the DPPH radical-scavenging ability of CEAX [36]. Therefore, the results demonstrated that the reaction environment had significant impact on the antioxidant ability of esterified FA and free FA. Such findings indicated that the results of the experiment based on physiological condition can have different results compared with the results in the in vitro experiment. Since physiological condition was mostly aqueous medium, the condition of our study was close to physiological condition to some extent. However, to acquire a complete understanding of FA antioxidant ability, further study can focus on its antioxidant activity in vivo.

**Figure 6:**
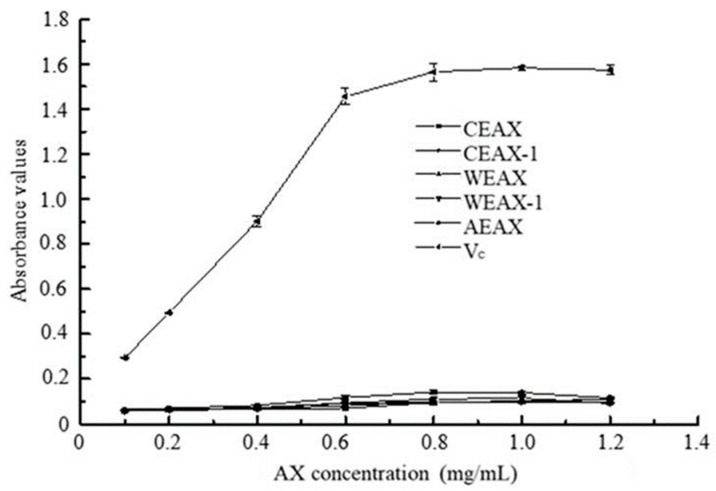
Reductive ability of arabinoxylans.

The reductive ability of AXs is important in the evaluation of their potential antioxidant capacity, in which the mechanism is totally based on electron transfer. However, no positive correlation was observed between the reductive ability of AXs and their concentration in our results (see Figure 6), which is inconsistent with the results reported by Rivas et al. [32] and Veenashri and Muralikrishna [37]. It has been reported that not all antioxidants reduced Fe^3+^ at a fast rate as anticipated; in addition, the reductive ability of polyphenols such as FA, tannic acid, and caffeic acid was slowly increasing even after several hours of reaction time [38]. Since FA is the major factor influencing the antioxidant capacity of AXs, the slow reaction rate may be one of the factors that limits the observation of the reductive ability of AXs.

Furthermore, the antioxidant capacity of FA has been reported to possibly inhibit the activity of α-glucosidase and α-amylase [9,10]. Zhang et al. (2016) believed that the antioxidant capacity of AXs can reduce postprandial oxidative stress that is associated with lower risks of diabetes [11]. Research studies indicated that AX antioxidant may contribute to their hypoglycemic activity. Thus, the present study further estimated the hypoglycemic activity of AXs.

### 3.3. Hypoglycemic Activity of AXs

**Figure 7:**
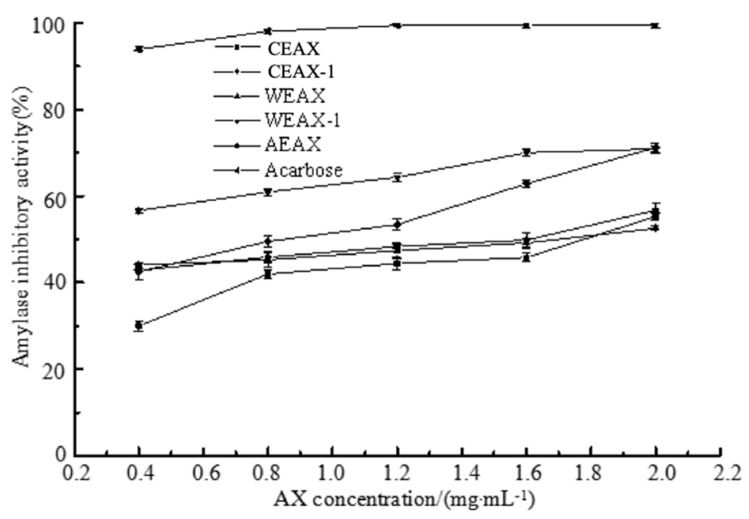
Inhibitory effects of arabinoxylans on the activities of α-amylase.

α-Amylase has the ability to break down long-chain carbohydrates, which can divide amylose into maltotriose and maltose as well as decompose amylopectin into maltose, glucose, and amylodextrin. Thus, the inhibition of α-amylase activity will contribute to the delay of increase in postprandial blood sugar levels [39]. The ability of AXs to inhibit α-amylase was determined, and the results are presented in Figure 7. The inhibition of α-amylase activity increased along with the increase in AX concentration, which confirmed the α-amylase inhibition ability of AXs. Moreover, the inhibition rate of α-amylase activity of alkali-extracted AXs was higher than of water- and enzyme-extracted AXs. FA content in AXs was reported to contribute to the α-amylase activity inhibition [9]. Malunga et al. also found that, compared with esterified FA, free FA had higher inhibition potency in rat intestine enzyme activity [10]. Therefore, alkali-extracted AXs demonstrated better inhibition of α-amylase activity possibly because of the higher amount of free FA contained in alkali-extracted AXs.

**Figure 8:**
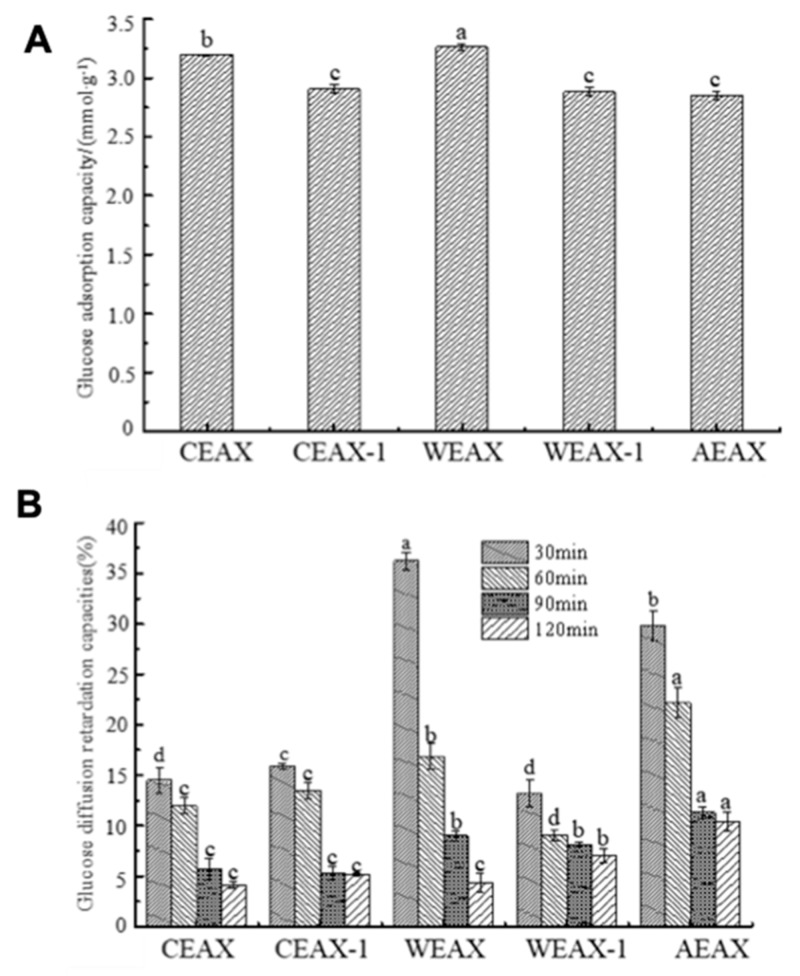
(**A**) Effect of arabinoxylans on the adsorption capacity for glucose and (**B**) effect of arabinoxylans on glucose dialysis retardation index. “a–d” indicates a significant in longitudinal, and “A–D” represents a significant in lateral.

The glucose absorption capacity of AXs was measured (see Figure 8A) to evaluate the hypoglycemic activity of AXs because it can lower glucose concentration absorbed by the small intestine. Our results showed that the glucose absorption capacity of water- and enzyme-extracted AXs was significantly higher than that of alkali-extracted AXs. It has been reported that viscosity affected the glucose absorption capacity of polysaccharides [40]. Esterified FA demonstrates a cross-linking effect that improves the gel-forming ability of AXs [41]. The amount of esterified FA was higher in water- and enzyme-extracted AXs; such a characteristic probably caused better glucose absorption capacity.

The GDRI is an important in vitro index to infer the effect of AXs on the delay in glucose absorption in the gastrointestinal tract [42]. The GDRI of AXs extracted through different methods is shown in Figure 8B. The adsorption process could reach a dynamic equilibrium and the glucose adsorption capacity of AXs could be close to saturation with the prolongation of dialysis time; therefore, the GDRI of all AXs was decreased over time. Moreover, the GDRI of WEAX was notably higher than that of CEAX (*p* < 0.05) because of the better solubility of WEAX [42]. Higher solubility possibly makes WEAX easy to interact with glucose, which leads to a higher glucose absorption capacity.

The results demonstrated that FA content of AXs plays an important role in their hypoglycemic activity. Esterified FA also exhibited a higher ability to elevate AX viscosity and inhibit α-amylase activity compared with free FA. Moreover, the MW and DS of AXs were able to affect the viscosity of AXs, thereby exerting effects on their hypoglycemic activity [7].

## 4. Conclusions

AXs extracted from triticale through different methods had inconsistent structural features that affected the antioxidant and hypoglycemic activities of AXs in vitro. FA played a significant role in antioxidant and hypoglycemic activities. Enzyme-extracted AXs contained higher levels of esterified FA that led to higher radical-scavenging activity. In contrast, alkali-extracted AXs contained higher levels of free FA content that led to a higher α-amylase inhibition ability. Both DS and MW were related to the antioxidant ability of AXs. Alkali-extracted AXs exhibited significant metal-chelating activity because of higher MW. Water-extracted AXs further demonstrated the highest glucose absorption capacity because of their good dissolvability. Therefore, AXs with higher esterified FA content and MW have higher antioxidant ability, whereas AXs with higher free FA level and solubility exhibit higher hypoglycemic activity.

## Figures and Tables

**Table 1:** Yield of arabinoxylans (AXs) extracted by different methods.

Samples	Yield (%)
CEAX	5.27 ± 0.09
CEAX-1	14.95 ± 0.17
WEAX	1.19 ± 0.17
WEAX-1	17.41 ± 0.13
AEAX	19.83 ± 0.16

Yield (%) was calculated as weight percentage of obtained AXs based on destarched triticale. AXs, arabinoxylans; CEAXs, complex enzyme-extracted AXs; CEAX-1, alkali-extracted AX from residue of complex enzyme extraction; WEAXs, water extractable AXs; WEAX-1, alkali-extracted AX from residue of water extraction; AEAX alkali extractable AXs.

**Table 2:** Monosaccharide components of AX extracted by different methods.

Samples	Arabinose (%)	Xylose (%)	Rhamnose (%)	Mannose (%)	Glucose (%)	Galactose (%)	Ara/Xyl
CEAX	18.19	73.57	n.d.	1.78	3.44	3.01	0.25
CEAX-1	55.90	36.83	n.d.	2.84	n.d.	4.43	1.52
WEAX	34.29	51.53	n.d.	2.30	7.29	4.59	0.67
WEAX-1	53.37	41.25	n.d.	n.d.	2.17	3.21	1.29
AEAX	48.18	42.33	n.d.	n.d.	5.90	3.58	1.14

All values were determined in duplicate. n.d., not detectable; Ara/Xyl, arabinose/xylose ratio; AXs, arabinoxylans; CEAXs, complex enzyme-extracted AXs; CEAX-1, alkali-extracted AX from residue of complex enzyme extraction; WEAXs, water extractable AXs; WEAX-1, alkali-extracted AX from residue of water extraction; AEAX, alkali extractable AXs.

**Table 3:** Molecular weight (MW) distribution of AX extracted by different methods.

Samples	Mw	Mn	Mw/Mn
CEAX	2.8314 × 10^4^	3744	7.56
CEAX-1	1.76876 × 10^5^	9259	19.10
WEAX	3.0730 × 10^4^	3312	9.28
WEAX-1	1.61989 × 10^5^	9274	17.47
AEAX	2.10638 × 10^5^	10,498	20.06

Mw, weight-average molecular weight; Mn, number-average molecular weight; AXs, arabinoxylans; CEAXs, complex enzyme-extracted AXs; CEAX-1, alkali-extracted AX from residue of complex enzyme extraction; WEAXs, water extractable AXs; WEAX-1, alkali-extracted AX from residue of water extraction; AEAX, alkali extractable AXs.

## References

[1] Döring C., Jekle M., Becker T. (2016). Technological and Analytical Methods for Arabinoxylan Quantification from Cereals. Crit. Rev. Food Technol..

[2] Snelders J., Dornez E., Delcour J.A., Courtin C.M. (2013). Ferulic Acid Content and Appearance Determine the Antioxidant Capacity of Arabinoxylanoligosaccharides. J. Agric. Food Chem..

[3] Malunga L.N., Beta T. (2015). Antioxidant capacity of arabinoxylan oligosaccharide fractions prepared from wheat aleurone using *Trichoderma viride* or *Neocallimastix patriciarum* xylanase. Food Chem..

[4] Malunga L.N., Izydorczyk M., Beta T. (2017). Effect of water-extractable arabinoxylans from wheat aleurone and bran on lipid peroxidation and factors influencing their antioxidant capacity. Bioact. Carbohydr. Diet. Fibre.

[5] Yuwang P., Sulaeva I., Hell J., Henniges U., Bohmdorfer S., Rosenau T., Chitsomboon B., Tongta S. (2018). Phenolic compounds and antioxidant properties of arabinoxylan hydrolysates from defatted rice bran. J. Sci. Food Agric..

[6] Bijalwan V., Ali U., Kesarwani A.K., Yadav K., Mazumder K. (2016). Hydroxycinnamic acid bound arabinoxylans from millet brans-structural features and antioxidant activity. Int. J. Biol. Macromol..

[7] Gemen R., De Vries J.F., Slavin J.L. (2011). Relationship between molecular structure of cereal dietary fiber and health effects: Focus on glucose/insulin response and gut health. Nutr. Rev..

[8] Christensen K.L., Hedemann M.S., Laerke H.N., Jorgensen H., Mutt S.J., Herzig K.H., Bach Knudsen K.E. (2013). Concentrated arabinoxylan but not concentrated beta-glucan in wheat bread has similar effects on postprandial insulin as whole-grain rye in porto-arterial catheterized pigs. J. Agric. Food Chem..

[9] Sales P.M., Souza P.M., Simeoni L.A., Magalhães P.O., Silveira D. (2012). α-Amylase Inhibitors: A Review of Raw Material and Isolated Compounds from Plant Source. J. Pharm. Pharm. Sci..

[10] Malunga L.N., Eck P., Beta T. (2016). Inhibition of Intestinal alpha-Glucosidase and Glucose Absorption by Feruloylated Arabinoxylan Mono- and Oligosaccharides from Corn Bran and Wheat Aleurone. J. Nutr. Metab..

[11] Zhang Z., Kong F., Ni H., Mo Z., Wan J.-B., Hua D., Yan C. (2016). Structural characterization, α-glucosidase inhibitory and DPPH scavenging activities of polysaccharides from guava. Carbohydr. Polym..

[12] Li W., Beta T., Sun S., Corke H. (2006). Protein characteristics of Chinese black-grained wheat. Food Chem..

[13] Sun Y., Cui S.W., Gu X., Zhang J. (2011). Isolation and structural characterization of water unextractable arabinoxylans from Chinese black-grained wheat bran. Carbohydr. Polym..

[14] Fadel A., Mahmoud A.M., Ashworth J.J., Li W., Ng Y.L., Plunkett A. (2018). Health-related effects and improving extractability of cereal arabinoxylans. Int. J. Biol. Macromol..

[15] Zhou S., Liu X., Guo Y., Wang Q., Peng D., Cao L. (2010). Comparison of the immunological activities of arabinoxylans from wheat bran with alkali and xylanase-aided extraction. Carbohydr. Polym..

[16] Chen Y., Xie M.Y., Wang Y.X., Nie S.P., Li C. (2010). Analysis of the monosaccharide composition of purified polysaccharides in Ganoderma atrum by capillary gas chromatography. Phytochem. Anal..

[17] Adriana M.O., Elizabeth C.M., Yolanda L.F., Agustín R.C., Jaime L.M., Patricia T.C., Alma C.M. (2013). Characterization of water extractable arabinoxylans from a spring wheat flour: Rheological properties and microstructure. Molecules.

[18] Apostolidis E., Lee C. (2010). In Vitro Potential of Ascophyllum nodosum Phenolic Antioxidant-Mediated α-Glucosidase and α-Amylase Inhibition. J. Food Sci..

[19] Ou S., Kwok K.C., Li Y., Fu L. (2001). In vitro study of possible role of dietary fiber in lowering postprandial serum glucose. J. Agric. Food Chem..

[20] Ak T., Gülçin I. (2008). Antioxidant and radical scavenging properties of curcumin. Chem. Interact..

[21] Sharma O.P., Bhat T.K. (2009). DPPH antioxidant assay revisited. Food Chem..

[22] Reis F.S., Martins A., Barros L., Ferreira I.C. (2012). Antioxidant properties and phenolic profile of the most widely appreciated cultivated mushrooms: A comparative study between in vivo and in vitro samples. Food Chem. Toxicol..

[23] Karnjanapratum S., Benjakul S. (2015). Antioxidative gelatin hydrolysate from unicorn leatherjacket skin as affected by prior autolysis. Int. Aquat. Res..

[24] Aguedo M., Fougnies C., Dermience M., Richel A. (2014). Extraction by three processes of arabinoxylans from wheat bran and characterization of the fractions obtained. Carbohydr. Polym..

[25] Hollmann J., Lindhauer M. (2005). Pilot-scale isolation of glucuronoarabinoxylans from wheat bran. Carbohydr. Polym..

[26] Maes C., Delcour J. (2002). Structural Characterisation of Water-extractable and Water-unextractable Arabinoxylans in Wheat Bran. J. Cereal Sci..

[27] Collins T., Gerday C., Feller G. (2010). Xylanases, xylanase families and extremophilic xylanases. FEMS Microbiol. Rev..

[28] Mzoughi Z., Abdelhamid A., Rihouey C., Le Cerf D., Bouraoui A., Majdoub H. (2018). Optimized extraction of pectin-like polysaccharide from *Suaeda fruticosa* leaves: Characterization, antioxidant, anti-inflammatory and analgesic activities. Carbohydr. Polym..

[29] Wang J., Hu S., Nie S., Yu Q., Xie M. (2016). Reviews on Mechanisms of In Vitro Antioxidant Activity of Polysaccharides. Oxidative Med. Cell. Longev..

[30] Jian L., Haifeng Z., Jian C., Wei F., Jianjun D., Weibao K., Junyong S., Yu C., Guolin C. (2007). Evolution of phenolic compounds and antioxidant activity during malting. J. Agric. Food Chem..

[31] Prior R.L., Wu X., Schaich K. (2005). Standardized Methods for the Determination of Antioxidant Capacity and Phenolics in Foods and Dietary Supplements. J. Agric. Food Chem..

[32] Rivas S., Conde E., Moure A., Domínguez H., Parajó J.C. (2013). Characterization, refining and antioxidant activity of saccharides derived from hemicelluloses of wood and rice husks. Food Chem..

[33] Foti M.C. (2015). Use and Abuse of the DPPH• Radical. J. Agric. Food Chem..

[34] Pristov J.B., Mitrović A., Spasojevic I. (2011). A comparative study of antioxidative activities of cell-wall polysaccharides. Carbohydr. Res..

[35] Izydorczyk M., Biliaderis C., Bushuk W. (1990). Oxidative gelation studies of water-soluble pentosans from wheat. J. Cereal Sci..

[36] Dervilly-Pinel G., Rimsten L., Saulnier L., Andersson R., Åman P. (2001). Water-extractable Arabinoxylan from Pearled Flours of Wheat, Barley, Rye and Triticale. Evidence for the Presence of Ferulic Acid Dimers and their Involvement in Gel Formation. J. Cereal Sci..

[37] Veenashri B., Muralikrishna G. (2011). In vitro anti-oxidant activity of xylo-oligosaccharides derived from cereal and millet brans—A comparative study. Food Chem..

[38] Ou B., Huang D., Hampsch-Woodill M., Flanagan J.A., Deemer E.K. (2002). Analysis of Antioxidant Activities of Common Vegetables Employing Oxygen Radical Absorbance Capacity (ORAC) and Ferric Reducing Antioxidant Power (FRAP) Assays: A Comparative Study. J. Agric. Food Chem..

[39] Cao C., Huang Q., Zhang B., Li C., Fu X. (2018). Physicochemical characterization and in vitro hypoglycemic activities of polysaccharides from *Sargassum pallidum* by microwave-assisted aqueous two-phase extraction. Int. J. Biol. Macromol..

[40] Vaugelade P., Hoebler C., Guillon F., Lahaye M., Duée P.-H. (2000). Non-starch polysaccharides extracted from seaweed can modulate intestinal absorption of glucose and insulin response in the pig. Reprod. Nutr. Dev..

[41] Chen Z., Li S., Fu Y., Li C., Chen D., Chen H. (2019). Arabinoxylan structural characteristics, interaction with gut microbiota and potential health functions. J. Funct. Foods.

[42] Rubio-Senent F., Rodríguez-Gutiérrez G., Muñoz A.L., Fernández-Bolaños J. (2015). Pectin extracted from thermally treated olive oil by-products: Characterization, physico-chemical properties, in vitro bile acid and glucose binding. Food Hydrocoll..

